# One-Year Survival after Cardiac Surgery in Frail Older People—Social Support Matters: A Prospective Cohort Study

**DOI:** 10.3390/jcm12144702

**Published:** 2023-07-15

**Authors:** Maria de Lurdes Castro, Marta Alves, Ana Luisa Papoila, Amália Botelho, José Fragata

**Affiliations:** 1 Anesthesiology Department, Hospital de Santa Marta, Centro Hospitalar Universitário Lisboa Central, Rua de Santa Marta, 50, 1169-024 Lisbon, Portugal; 2 Epidemiology and Statistics Unit, Research Centre, Centro Hospitalar Universitário Lisboa Central, Rua Jacinta Marto, 1169-045 Lisbon, Portugal; marta.l.alves@gmail.com (M.A.); apapoila@hotmail.com (A.L.P.); 3 Centre of Statistics and Its Applications (CEAUL), Faculty of Sciences, University of Lisbon, 1749-016 Lisbon, Portugal; 4 Independent Researcher, Lisbon 1400, Portugal; mariasmsbotelho@gmail.com; 5 Cardiothoracic University Clinic and Department, Hospital de Santa Marta, Centro Hospitalar Universitário Lisboa Central, Rua de Santa Marta, 50, 1169-024 Lisbon, Portugal; jigfragata@gmail.com

**Keywords:** survival, older people, cardiac surgery, frailty, depression, social support, EuroSCORE II, pneumonia, re-intervention

## Abstract

There are increasing rates of cardiac surgery in the elderly. Frailty, depression, and social vulnerability are frequently present in older people, and should be considered while assessing risk and providing treatment options. We aimed to analyse the impact of clinically relevant variables on survival at one year, and identify areas of future intervention. We performed a prospective cohort study at a University Hospital, with a sample of 309 elective cardiac surgery patients 65 years old and over. Their socio-demographic and clinical variables were collected. Frailty prevalence was 61.3%, while depression was absent in the majority of patients. Mortality was 1.6% and 7.8% at 30 days and 12 months, respectively. After Kaplan–Meier analysis, severe frailty (*p* = 0.003), severe depression (*p* = 0.027), pneumonia until 30 days (*p* = 0.014), and re-operation until 12 months (*p* = 0.003) significantly reduced survival, while social support increased survival (*p* = 0.004). In the adjusted multivariable Cox regression model, EuroSCORE II (HR = 1.27 [95% CI 1.069–1.499] *p* = 0.006), pneumonia until 30 days (HR = 4.19 [95% CI 1.169–15.034] *p* = 0.028), re-intervention until 12 months (HR = 3.14 [95% CI 1.091–9.056] *p* = 0.034), and social support (HR = 0.24 [95% CI 0.079–0.727] *p* = 0.012) explained time until death. Regular screening for social support, depression, and frailty adds relevant information regarding risk stratification, perioperative interventions, and decision-making in older people considered for cardiac surgery.

## 1. Introduction

Cardiac surgery mortality has dropped in the last twenty years as a result of both medical and technical evolution, as well as proper risk stratification and patient selection [1,2]. However, a diverging trend was observed during the COVID-19 pandemic in 2020–2021, in which crude mortality increased along with a reduction in the number of elective cardiac surgery procedures performed, and an increase in the proportion of interventional cardiology procedures [3,4].

Risk stratification tools, such as EuroSCORE and the Society of Thoracic Surgeons (STS) Score, have served to better inform both healthcare professionals and patients about the risk of a given procedure, and also to assess the quality of cardiac surgical services.

EuroSCORE is a global risk model for cardiac surgery while the STS score is available for specific cardiac surgeries. A frequently referred limitation of both these models is their limited accuracy for predicting mortality in patients over 80 years old [5]. This is particularly relevant considering the projected increase of about 60% in people 75–84 years old in Europe [6], and estimates of about 20% of individuals who are 75 years old and older undergoing any kind of surgery by 2030. Older people frequently present multimorbidity and multifactorial health problems, and geriatric medicine has renewed the relevance of considering all human functioning dimensions when delivering care. Frailty is an increasingly used concept in geriatric medicine and is generally accepted as a state of increased vulnerability to adverse outcomes after a stressor event. The pathophysiology involves low-level chronic inflammation, impaired immunity, neuroendocrine dysregulation, and metabolic alterations, namely sarcopenia. It is probably the result of multiple subcellular events that are secondary to lifestyle and environmental factors combined with genetic susceptibility. There is evidence of a higher prevalence of frailty (from 42% to 60%) in cardiovascular patients that are 65 years old and over, when compared to the same-age general population (around 10%), suggesting shared risk factors and pathophysiology for these conditions [7].

Several tools in different settings have been developed to address the issue of frailty impacts on outcomes, while also considering their ease of use. Multiple studies have shown an increase in the predictive power of the usual perioperative risk indexes when a frailty tool is added, both in cardiac and non-cardiac surgery [8,9]. The Edmonton Frail Scale ©has been designed as a bedside screening tool to be used by non-geriatricians [10] and has been used in perioperative settings [11]. Recently, the Portuguese version of the Edmonton Frail Scale© was shown to be valid and reproducible for use in cardiac surgery patients [12]. This allowed us to use it in this research, as we intended to screen for frailty using a valid and reproducible multidimensional tool.

Depression frequently coexists with frailty, their overlapping being greater in higher grades of both conditions [13]. Depression is associated with increased cardiovascular risk burden, and management of depression through psychological interventions may be associated with better outcomes in cardiovascular disease patients [14]. There is also a relevant interplay between these conditions and social context. Depressive symptomatology, social isolation, and feelings of loneliness are progressively higher when frailty increases [15]. Social frailty is a complex construct, involving different domains related to social needs fulfilment [16]. Social isolation and social support are predictors of cardiovascular disease incidence and mortality [17], while social networks are associated with longer life expectancy [18]. Possible mediators of this effect are the influence of health-related behaviors, the provision of effective and perceived social support, access to resources, and impact on the immune system from pathogen exchange, as well as pro-inflammatory cytokine [19] reduction.

Portuguese clinical research addressing perioperative outcomes in older people is scarce, which led us to perform a prospective longitudinal study of the outcomes of older cardiac surgery patients. We aimed to estimate the prevalence of frailty, depression, and cognitive impairment before cardiac surgery and to analyze the impact of pre-, intra-, and postoperative variables on survival of older patients one year after elective cardiac surgery.

## 2. Methods

We conducted a prospective cohort study of elective cardiac surgery patients from Centro Hospitalar Universitário de Lisboa Central (CHULC). Patients treated in Clínica Universitária de Cirurgia Cardiotorácica, CHULC, are referred from this hospital center and from other health institutions according to the national referral network. Elective cardiac surgery patients’ recruitment took place in preoperative anaesthesia consultation between July 2016 and November 2018, and follow-up ended in February 2020. Data were collected prospectively until the end of follow-up.

A sample of consecutive patients 65 years old and above, admitted for elective isolated valve, isolated coronary artery bypass graft (CABG), or combined valve and coronary artery bypass surgeries, evaluated in preoperative anaesthesia consultation by the main investigator, were selected. Exclusion criteria were complex aortic arch procedures, urgent or redo surgery, critical preoperative state, and hearing or visual impairment preventing patients from understanding or performing tasks required by the study protocol.

In our institution, cardiac surgery patients are usually followed up for one year, except for those who explicitly ask to end surgical follow-up earlier and continue to be followed by their assistant cardiologist, for their own convenience—usually closer to their residence. Portuguese versions of validated screening tools for frailty (Edmonton Frail Scale©), depression (Geriatric Depression Scale with 30 and 15 items), and cognitive status (Mini-Mental State Examination and Clock Drawing Test as part of Edmonton Frail Scale©) were administered preoperatively (scales used available in Supplementary Materials). Socio-demographic and clinical variables were prospectively collected from electronic and paper records in up to three visits after surgery, according to their scheduled surgical postoperative consultations at 3, 6, and 12 months. All data were reviewed and entered by the main investigator in standardized case report forms and in an electronic study database. Data regarding survival, which were collected from an administrative national registry, were available for all the enrolled patients. The complete list of variables is depicted in Table 1. The primary outcome was survival at 12 months.

### Statistical Analysis

The sample size was calculated regarding the primary objective of frailty prevalence estimation. Accordingly, the formula [20] that was used considered a level of confidence of 95%, an expected prevalence of 15% [5], and a precision of 4% (corresponding to the sampling error or deviation between the sampling prevalence from the population prevalence) was considered, thereby achieving a sample size of 306 patients. Epi Info™ version 7 (CDC—Center for Diseases Control and Prevention, Atlanta, GA, USA) was used to obtain the sample size [21].

The characteristics of study participants were described with frequencies (percentages) and with mean (standard deviation) or median (min–max) as appropriate. Student’s *t*, Chi-Square and Mann–Whitney tests were used when required.

For survival analysis, age was treated as quantitative variable, number of school years as categorical variable (up to 4 years of schooling and more than 4 years); civil status as categorical variable (married or all other); ASA as categorical variable (III or IV); ESII, STS M, and STS MM as quantitative variables; EFS as categorical variable (Fit to Moderate Frailty versus Severe Frailty); GDS30 as categorical variable (Lack of Depression to Mild Depression versus Severe Depression); GDS15 and MMSE as quantitative variables; performed surgery and intraoperative adverse events as categorical variables; cardiopulmonary bypass, aortic cross-clamping, and mechanical ventilatory support duration as quantitative variables; and delirium, adverse outcomes at 30 days or during hospital stay (standardized according to STS version 2.61 Appendix A), and re-operation until 12 months as categorical variables.

Kaplan–Meier curve estimates were plotted for categorical variables and Log-rank tests used. Univariable Cox regression was performed and those variables having a *p*-value ≤ 0.25 were candidates for the multivariable Cox regression models. A stepwise forward method was used and two final models, with and without the variable of social support, were compared using a likelihood ratio test. A level of significance α = 0.05 was considered. Statistical analysis was performed using SPSS^®^ (Statistical Package for the Social Sciences) version 28 (IBM Corp. Released 2021. IBM SPSS Statistics for Windows, version 28.0. Armonk, NY: IBM Corp, USA) and R^®^ software (R Core Team 2023). R: A Language and Environment for Statistical Computing. R Foundation for Statistical Computing, Vienna, Austria. URL https://www.R-project.org/. (accessed on 24 February 2023).

This study was approved by NOVA Medical School/Faculdade de Ciências Médicas and Centro Hospitalar Universitário de Lisboa Central (CHULC) prior to initiation. After informing study participants about the nature and goals of the study, consent for data collection was obtained at time of enrolment. All data were anonymized.

STROBE statement on reporting of cohort studies was followed (URL https://www.equator-network.org/) (accessed on 6 June 2023)

## 3. Results

**Figure 1:**
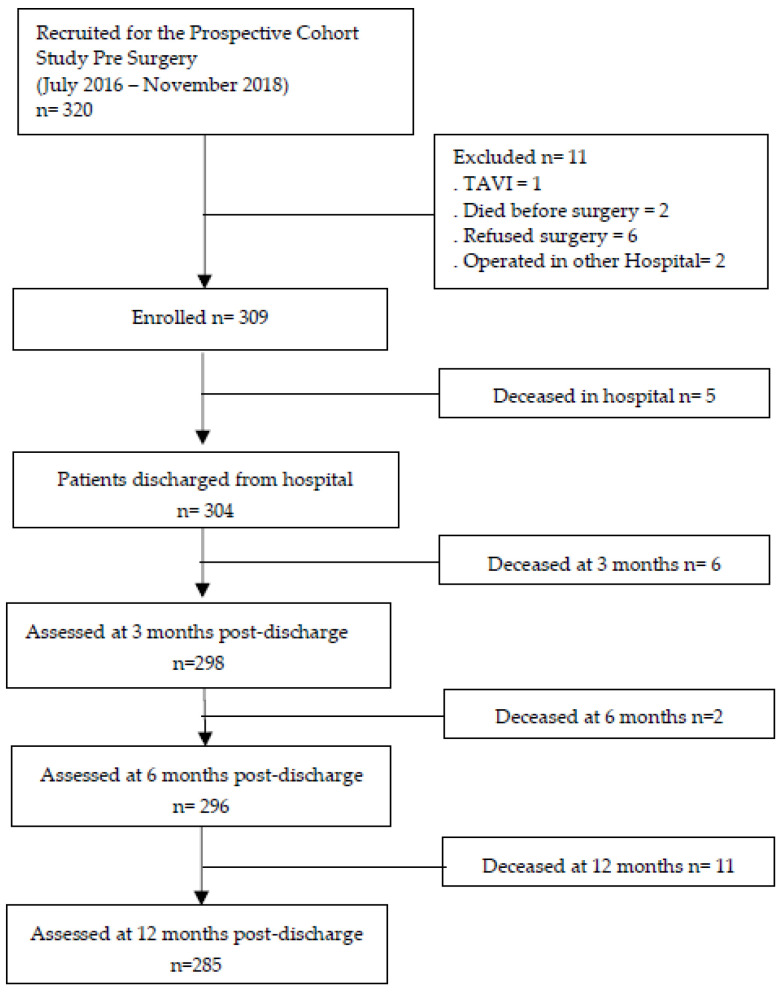
Flowchart of enrolment and follow-up. Legend: TAVI—Transfemoral Aortic Valve Implantation.

Figure 1 depicts the flowchart of enrolled patients for the prospective cohort study.

The study population comprised 309 patients, with 54.4% men. The mean age was 74.4, ranging from 65 to 88 years, with 19.1% being 80 years old or more. Most patients were married (65.4%), had social support (94.8%), and completed one to four schooling years (68.6%). ASA physical status was either III (62.1%) or IV (37.9%). The median for ESII was 1.85 and for STS M it was 1.84. STS Morbimortality median was 11.87 (Table 2). Regarding frailty scores in EFS, 11.7% of patients were considered fit, 27.0% vulnerable, and the remaining 61.3% were frail, with 14.3% scoring for severe frailty. Most patients did not score for depression according to either GDS30 (64.4%) or GDS15 (67.6%), with 4.5% scoring for severe depression in GDS30. Considering preoperative MMSE, 18.9% of patients scored for cognitive deficit, scoring less than 26 in 30—according to Freitas et al.’s [22] normative data published in 2014 for the Portuguese population that is 65 years and older and has 1 to 4 schooling years—while 39.2% scored for major errors in the Clock Drawing Test, the Cognition Domain tool that included in the Edmonton Frail Scale (Table 2).

Isolated valve surgery was performed in 59.2%, CABG in 25.9%, and combined procedures in 14.9% of patients. Intraoperative adverse events occurred in 1.6%.

Surgery with cardiopulmonary bypass occurred in 80.3% of patients. Cardiopulmonary bypass (CPB) median time was 89 min and aortic cross-clamping median time was 69 min.

Postoperative mechanical ventilation median time was 8 h. Delirium during hospital stay occurred in 27.2% of patients. At 30 days, sternal infection occurred in 1.3%, myocardial infarction in 1.3%, re-operation in 2.9%, pneumonia in 3.9%, renal failure in 4.2%, and stroke in 4.5%. A composite of at least one major adverse postoperative outcome occurred in 14.2% of patients. Re-operation until one year after first surgery occurred in 6.8% of patients.

Observed number of deaths at 30 days, 90 days, and 12 months was 5 (1.6%), 11 (3.6%), and 24 (7.8%), respectively.

After analyzing socio-demographic and clinical variables according to sex, mean age was significantly higher for women, the median number of school years was significantly lower for women, and a larger proportion of women were widowed. No difference was found regarding social support between men and women. For all the risk scores considered, the medians were significantly higher for women. Regarding frailty and depression scores, women had statistically significant higher scores, and for MMSE, there were statistically significant lower scores—although irrelevant from a clinical point of view (Table 3).

### 3.1. Kaplan–Meier Survival Curve Estimates at 365 Days

**Figure 2:**
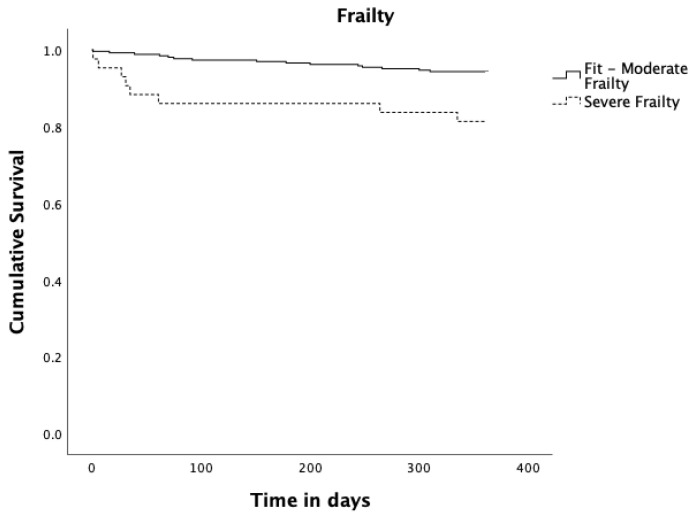
Kaplan–Meier survival curves estimates for Frailty. Solid black line represents Fit to Moderate Frailty (n = 266; 16 events and 250 censored observations). Dashed black line represents Severe Frailty (n = 43; 8 events and 35 censored observations), *p* = 0.003.

**Figure 3:**
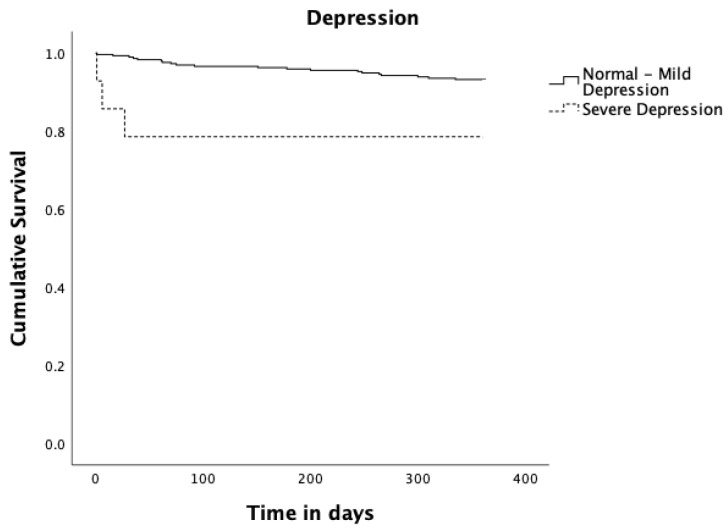
Kaplan–Meier survival curves estimates for Depression. Solid black line represents Normal to Mild Depression (n = 295; 21 events and 274 censored observations). Dashed black line represents Severe Depression (n = 14; 3 events and 11 censored observations), *p* = 0.027.

More relevant Kaplan–Meier analysis results are shown (Figure 2 and Figure 3). No significant difference was found in survival between men and women (*p* = 0.216), between married and all other civil statuses (*p* = 0.453), between having up to 4 schooling years or more (*p* = 0.311), nor when comparing patients in isolated CABG, isolated valve, and combined CABG and valve surgery groups (*p* = 0.271).

Regarding frailty measured by the binary Edmonton Frail Scale (fit to moderate frailty vs severe frailty), patients who scored preoperatively for severe frailty had a statistically significant reduction in survival compared to patients who scored preoperatively from fit to moderate frailty (*p* = 0.003). Concerning depression measured by GDS30 as binary (normal to mild depression vs severe depression), patients scoring for severe depressive symptoms had a significant reduction in survival (*p* = 0.027). Patients diagnosed with pneumonia up to 30 days after surgery had a significantly reduced survival (*p* = 0.014). Those who underwent re-operation up to 365 days after surgery had a significantly reduced survival (*p* = 0.003), and finally patients with social support had a significant increase in survival (*p* = 0.004).

### 3.2. Cox Regression

Univariable Cox regression results for the candidate variables to the multivariable analysis are shown in Table 4. All the remaining risk factors considered in this study obtained *p*-values > 0.25. Though both frailty (fit to moderate frailty versus severe frailty) and GDS30 (normal to mild depression vs severe depression) were considered as candidates for the multivariable Cox regression analysis, they did not remain in the final model after adjusting by other candidate variables. Final models (1 and 2), with and without the variable social support, are presented in Table 5. The model including the variable of social support is the model with the best performance, with a significant likelihood ratio test (*p* = 0.030) when compared with the simpler model without this variable.

The analysis of model 2 shows that for each increase of one unit in ESII, the risk of death until 12 months increased by about 27%. Pneumonia up to 30 days after surgery increased the risk of death about four times and re-intervention up to 365 days increased the risk of death about three times. Social support was a protective factor, reducing the risk of dying up to one year after surgery by 76%.

## 4. Discussion

In our cohort, the mean age was 74.4 years old, with a similar proportion of male and female patients and with 19.1% of patients being 80 or more years old, which is consistent with the demographic trends reported [1] of an increased proportion of patients above 80 years old in the cardiac surgery population. Preoperative frailty measured by the Edmonton Frail Scale (EFS) was present in 61.3% of patients, with 14.3% scoring for severe frailty. This prevalence is higher than in other recent reported longitudinal studies [11,23], which might be related to both selection bias and the tool used to measure frailty. Though severe depression, as screened by GDS30, was present in a minority (4.5%) of patients, it is still relevant since these patients had a reduced survival. Regarding preoperative cognitive status, 18.9% scored for cognitive deficit in the Mini-Mental State Examination (MMSE), while a higher proportion of 39.2% had major errors in the Clock Drawing Test (CDT)—the cognition domain test included in the Edmonton Frail Scale—indicating possible cognitive deficit. Preoperative cognitive deficit prevalence measured by MMSE is in accordance with another published study [24]. Though MMSE is still considered the gold standard screening tool for dementia, it can fail to detect executive dysfunction, which is frequently present in the initial stages of Alzheimer’s disease (AD) and vascular dementia, the two most common forms of dementia present in cardiovascular disease patients [25]. Another limitation of MMSE is its ceiling effect in individuals with high premorbid intelligence and education, leading to false negatives. On the other hand, CDT allows us to evaluate verbal understanding, memory, abstract thinking, planning, concentration, and visuo-constructive skills, though controversies remain about the level of education effect on performance. CDT can therefore be a means of detecting early cognitive decline in AD and vascular dementia, possibly before MMSE. The differences found in our population when applying these two tools may relate to the proportions of patients exhibiting dementia in different stages, but we cannot exclude an education effect in the results of CDT, at least to some degree, given that 68.6% of patients had between one and four schooling years [26].

The majority of our patients underwent isolated valve surgery, as reported by other recently published longitudinal studies in cardiac surgery and national series [1,23].

We found statistically significant differences in baseline characteristics between men and women: higher mean age, lower number of schooling years, and a higher proportion of widows among women, as well as higher median ESII, STS M, STS MM, GDS30, GDS15, and EFS preoperative scores, which are consistent with other studies and a recently published meta-analysis [23,27]. Nevertheless, no significant difference was found in survival when comparing men and women. Observed mortality at 30 days was 1.6%, which is lower than predicted by either ESII or STS M. Mortality at one year was lower than observed in other studies [9,22]. Relatively lower median ESII and STS M scores in our cohort may have contributed to this result, as well as the high percentage of frail patients (61.3%) treated in our center. There is evidence supporting frail patients’ results from cardiac surgery are associated with the hospital’s volume of frail patients treated, possibly owing to the faster recognition and treatment of complications in larger volume hospitals [28].

Frailty (EFS) and depression (GDS30) as binary variables, social support, pneumonia up to 30 days, and re-intervention up to 365 days were shown to be statistically significant after Kaplan–Meier survival analysis. In our cohort, patients scoring for severe frailty had a reduced survival, but no difference was found in survival between patients in other categories of the Edmonton Frail Scale—from fit to moderate frailty. We consider this to be an argument for the use of quantitative and multidomain measures instead of “eyeball-ing” and qualitative measures of frailty when evaluating patients proposed for cardiac surgery. This allows us to grade frailty, better inform the patient and team about the risk–benefit ratio of the intended intervention, and address specific vulnerabilities prior to surgery in order to reduce frailty. There is evidence [29] that targeted multidomain interventions allow the transition between different states in the fitness–frailty continuum, and as such, a larger number of patients could seize the advantages of cardiac surgery when frailty is previously reduced by a pre-habilitation program [30].

Patients scoring for severe depression had a reduced survival. This is in accordance with existing evidence, and both the European Association of Preventive Cardiology Position paper [31] and the American Heart Association [32] recommend managing psychosocial variables, namely depression, in patients with cardiovascular disease, as a means of improving outcomes and supporting the inclusion of strategies to reduce anxiety and depression in pre-habilitation programs.

Though patients with severe frailty and severe depression had a statistically significant reduction in survival throughout the follow-up period, and both variables were therefore candidates for the multivariable Cox regression, neither of them were a contributing variable to time until death when their effect was adjusted for other candidate variables. The best fitted model included ESII, pneumonia, and re-intervention up to 365 days as risk factors, and social support as a protective factor. Interestingly, in our cohort, ESII, one of the scores used to predict risk at 30 days for an individual patient before cardiac surgery, was valuable as a determinant variable for explaining time until death at 365 days, which is in contrast with other similar studies [9].

Re-operation, pneumonia, and social support may be viewed as factors amenable to intervention throughout the perioperative period.

Re-operation incidence varies widely between centers. It may be due to surgical re-exploration in the context of bleeding or surgical site infection. Known risk factors for bleeding requiring surgical exploration are EuroSCORE, preoperative hematocrit, and CPB duration [33]. Patient blood management in cardiac surgery guidelines have focused on preoperative hemoglobin optimization, fibrinogen dosing, and platelet function testing in patients at high risk of bleeding, tranexamic acid use in CABG patients, and viscoelastic point-of-care tests throughout the perioperative period [34]. Regarding surgical site infection, it may be superficial, either in the sternum or from the harvesting area, or deep (mediastinitis and endocarditis); the risk factors include female sex, advanced age, diabetes mellitus, chronic obstructive pulmonary disease, obesity, the use of bilateral internal mammary arteries, prolonged CPB time, re-exploration, blood product transfusion, and prolonged ventilatory support and ICU stay [35]. Preventive measures include optimizing patients’ premorbid conditions—namely correcting hypoalbuminemia, optimizing glycemia, smoking cessation, chest physiotherapy, and treating all sources of extra-thoracic infection before surgery—screening of *S. aureus* carriers and nasal mupirocin use, prophylactic antibiotic at 30 to 60 min prior to surgery, use of a chlorhexidine-alcohol-based solution for skin preparation, and removal of operative wound dressing after 48 h [35,36].

Re-operation incidence reduction requires awareness of these inter-related risk factors and the adoption of a bundle of measures, some of which are already implemented in our center.

Pneumonia increased the risk of death by four times; postoperative pneumonia incidence varies widely between centers, being consistently associated with worse outcomes. Several risk factors have been identified (advanced age, chronic obstructive pulmonary disease, chronic kidney disease, anemia, diabetes mellitus, CPB time, transfusion, and mechanical ventilation length), as well as preventive measures. Among the effective preventive measures are inspiratory muscle training and physiotherapy, oral care, subglottic secretions drainage, lung protective ventilation and recruitment maneuvers, effective multimodal opioid-sparing analgesia, early extubation, early mobilization and progressive ambulation, enteral feeding, selective digestive decontamination, proper use of non-invasive ventilation and tracheostomy, and microbiological surveillance [37].

Post-operative adverse outcomes are frequently interrelated, and it seems logical that full adherence to multimodal Enhanced Recovery After Surgery (ERAS) guidelines, along with definition of fast-track circuits in cardiac surgery patients, will probably further ameliorate the perioperative course, and reduce adverse outcomes, as has happened in other types of surgeries [36].

The only protective factor for survival identified in our cohort was social support. There is substantial evidence associating both lack of social support itself and of social support perception to worse outcomes in brain and cardiovascular health, such as incident coronary artery disease, incident congestive heart disease mortality, incident and recurrent stroke, and dementia and cognitive impairment [17,32]. On the other hand, existing social support and social support perception are associated with improved survival in oncological and cardiovascular disease [38]. The probable mediators are behavioral, psychological, and physiological, which affect each other [32]. Behavioral factors are related to a healthier diet and lifestyle (less alcohol and tobacco use, less sedentarism) and increased medication adherence in people with social support. Psychological and physiological factors are related to anxiety, loneliness and depression, and increased levels of pro-inflammatory biomarkers (IL-6 and CRP) in patients that perceive themselves as isolated. People with effective social support have reduced levels of pro-inflammatory cytokines, translating into less allostatic load—the wear and tear on the body accumulated by repeated stress exposure. Questions remain regarding the mechanisms underlying social support’s impact on survival. Nevertheless, it seems plausible to include this aspect in frail older surgical patients’ evaluation and management. Interventions to reduce social isolation showing most promise in older population are group physical activities (resistance exercise and walking). There is conflicting evidence about the benefit of mindfulness-based programs for depression, anxiety, and mild cognitive impairment in older populations. Some studies reported positive clinical effects, which are associated with a reduction in IL-6 and salivary cortisol levels [39], increased cerebral blood flow and brain function connectivity—measured by functional and structural MRI studies—and increased thickness in gray matter in areas related to executive function and emotional regulation [40], while others failed to demonstrate benefit.

Social and family context evaluations are routinely performed in the nursing consultation before surgery in our institution. Patients proposed for cardiac surgery are asked about their reference person, carer, co-inhabitants, and effective family and/or social support. Identified needs are addressed in a timely manner in coordination with the hospital and primary care social assistance services. Though this study was not designed to specifically assess this intervention, it is plausible to consider it might have impacted survival.

In conclusion, we found a high prevalence of frailty in our cohort. After Kaplan–Meier analysis, a statistically significant reduced survival was observed in patients scoring for severe frailty and severe depression. In the Cox multivariable regression, increasing ESII, occurrence of pneumonia at 30 days, and re-intervention until 365 days increased risk of death, while social support increased survival. The inclusion of regular frailty and depression screening, as well as the assessment of social support effectiveness and perception, in addition to standard risk scores, may add value in cardiac surgery patients’ risk stratification based on our results. Besides allowing for the identification of patients at the highest risk levels, in order to address their specific vulnerability domains, this screening offers the possibility of discussing available treatment options centered around patient values and context. The full adherence to ERAS guidelines and designing of a multidimensional tailored pre-habilitation program, with a focus on the identified risk factors that impact survival, has the potential to further improve mid- and long-term outcomes in our population.

## 5. Limitations

We performed a prospective cohort study that proved to be valuable in identifying factors related to survival at one year, which are amenable to intervention. However, the external validity of our results is limited given that it is a single-center observational study and is prone to selection bias related to the included participants. A multicenter study could possibly identify other factors impacting older patients’ survival after cardiac surgery.

## Figures and Tables

**Table 1:** Complete list of variables.

	Variables	
Preoperative	Intraoperative	Postoperative
Age	Performed surgery	Mechanical ventilatory support (hours)
Sex	Cardiopulmonary bypass (CPB) (minutes)	Delirium during first 5 days of hospital stay assessed with CAM (Confusion Assessment Method)
Civil status	Cross-clamping (minutes)	Stroke *
Social Support	Occurrence of adverse events	Myocardial Infarction *
Number of school years		Sternum Infection *
EuroSCORE II (ESII)		Re-Operation *
STS Mortality (STS M)		Renal Failure *
STS Morbimortality (STS MM)		Pneumonia *
ASA (American Society of Anesthesiologists) physical state		Composite of events signed with *
Edmonton Frail Scale (EFS)		
Geriatric Depression Scales with 30 (GDS30) and 15 (GDS15) items		Re-operation up to 12 months after the first surgery
Mini-Mental State Examination (MMSE)		

Legend: STS—Society of Thoracic Surgeons; * as defined in STS version 2.61 Appendix A.

**Table 2:** Characteristics of participants for the entire cohort and stratified according to status at 12 months.

Characteristics	Overall (n = 309)	Alive at 12 Months (n = 285)	Dead at 12 Months (n = 24)
Age, mean (SD)	74.4 (5.9)	74.4 (5.9)	75.1 (6.1)
Sex, n (%)			
Male	168 (54.4)	152 (53.3)	16 (66.7)
Female	141 (45.6)	133 (43.7)	8 (33.3)
Civil Status, n (%)			
Married	202 (65.4)	188 (66.0)	14 (58.3)
Single	10 (3.2)	8 (2.8)	2 (8.3)
Divorced	13 (4.2)	13 (4.6)	0 (0.0)
Widow	84 (27.2)	76 (26.7)	8 (33.3)
Social Support, n (%)			
Yes	292 (94.8)	273 (95.8)	20 (83.3)
No	16 (5.2)	12 (4.2)	4 (16.7)
Number of complete school years, n (%)			
1 to 4 years	212 (68.6)	173 (60.7)	12 (50.0)
5 to 9 years	49 (15.9)	44 (15.4)	5 (20.8)
10 to 12 years	32 (10.4)	29 (10.2)	3 (12.5)
>12 years	16 (5.2)	39 (13.7)	4 (16.7)
ASA physical status, n (%)			
III	192 (62.1)	180 (63.2)	12 (50.0)
IV	117 (37.9)	105 (36.8)	12 (50.0)
ESII, median (min–max)	1.85 (0.61–11.04)	1.76 (0.61–11.04)	2.41 (1.08–9.71)
STS M, median (min–max)	1.84 (0.34–11.36)	1.76 (0.34–11.36)	2.34 (0.74–9.14)
STS MM, median (min–max)	11.87 (3.33–57.06)	11.69 (3.33–57.06)	15.11 (6.60–33.96)
EFS (0–17), median (min–max)	6 (0-13)	6 (0-13)	6 (3–10)
Fit (0–3), n (%)	35 (11.3)	34 (11.9)	1 (4.2)
Vulnerable (4–5), n (%)	84 (27.2)	76 (26.7)	8 (33.3)
Mild Frailty (6–7), n (%)	86 (27.8)	80 (28.1)	6 (25.0)
Moderate Frailty (8–9), n (%)	61 (19.7)	60 (21.1)	1 (4.2)
Severe Frailty (+10), n (%)	43 (13.9)	35 (12.3)	8 (33.3)
GDS30 (0–30], median (min–max)	8 (0–28)	8 (0-28)	9 (2–18)
Normal (0–10), n (%)	199 (64.4)	185 (64.9)	14 (58.3)
Mild Depression (11–20), n (%)	96 (31.1)	89 (31.2)	7 (29.2)
Severe Depression (21–30), n (%)	14 (4.5)	11 (3.9)	3 (12.5)
GDS15 (0–15), median (min–max)	3 (0–13)	3 (0–13)	3 (0–9)
Normal (0–4), n (%)	209 (67.6)	196 (68.8)	13 (54.2)
Mild Depression (5–8), n (%)	79 (25.6)	72 (25.3)	7 (29.2)
Moderate Depression (9–11), n (%)	17 (5.5)	14 (4.9)	3 (12.5)
Severe Depression (12–15), n (%)	4 (1.3)	3 (1.1)	1 (4.2)
MMSE (0–30), median (min–max)	28 (14-30)	28 (14–30)	28 (20–30)
Cognitive Impairment (MMSE < 26)	46 (18.9)	44 (19.5)	2 (11.8)
Major Errors in Clock Drawing Test	121 (39.2)	114 (40.0)	7 (29.2)
Performed Surgery, n (%)			
CABG	80 (25.9)	77 (27.0)	3 (12.5)
Isolated Valve	183 (59.2)	167 (58.6)	16 (66.7)
Combined CABG and Valve	46 (14.9)	41 (14.4)	5 (20.8)
CPB (minutes), median (min–max)	89 (0–478)	89 (0–321)	108 (0–478)
Cross-clamping (minutes), median (min–max)	69 (0–303)	68 (0-262)	83.0 (0–303)
Intraoperative adverse events, n (%)	5 (1.6)	3 (1.1)	2 (8.3)
Mechanical ventilation (hours), median (min–max)	8 (0–260)	8 (0–260)	10 (0–48)
Delirium, n (%)	84 (27.2)		
Stroke *, n (%)	14 (4.5)	13 (4.6)	1 (4.2)
Myocardial Infarction *, n (%)	4 (1.3)	3 (1.1)	1 (4.2)
Sternum Infection *, n (%)	4 (1.3)	2 (0.7)	2 (8.3)
Re-Operation *, n (%)	9 (2.9)	6 (2.1)	3 (12.5)
Renal failure *, n (%)	13 (4.2)	10 (3.5)	3 (12.5)
Pneumonia *, n (%)	12 (3.9)	9 (3.2)	3 (12.5)
Composite of *, n (%)	44 (14.2)	36 (12.6)	8 (33.3)
Re-operation until 365 days, n (%)	21 (6.8)	16 (5.6)	5 (20.8)

Legend: ASA: American Society of Anesthesiologists; ESII: EuroSCORE II; STS M: Society of Thoracic Surgeons Mortality; STS MM: Society of Thoracic Surgeons Morbimortality; EFS: Edmonton Frail Scale; GDS 30: Geriatric Depression Scale 30 items; GDS15: Geriatric Depression Scale 15 items; MMSE: Mini-Mental State Examination; CABG: Coronary Artery Bypass Graft; CPB: Cardiopulmonary Bypass. * as defined in STS version 2.61 Appendix A.

**Table 3:** Preoperative Socio-demographic Scores and Scales according to sex.

Characteristics	Female (n = 141)	Male (n = 168)	*p*-Value
Age, mean (SD)	75.63 (5.95)	73.39 (5.65)	<0.001
Number of complete school years, mean (SD)	4.93 (3.58)	5.96 (4.25)	0.01
Widows, n (%)	64 (45.4)	20 (11.9)	<0.001
Social Support, n (%)	134 (95.0)	159 (94.6)	0.877
ESII, median (min-max)	2.10 (0.79–6.95)	1.47 (0.61–11.04)	<0.001
STS M, median (min-max)	2.41 (0.46–6.62)	1.29 (0.34–11.36)	<0.001
STS MM, median (min-max)	13.37 (4.22–48.64)	10.35 (3.33–57.06)	<0.001
EFS, median (min-max)	7 (1–13)	5 (0–13)	<0.001
GDS30, median (min-max)	10 (1–25)	7 (0–28)	<0.001
GDS15, median (min-max)	4 (0–13)	2(0–13)	<0.001
MMSE, median (min-max)	27.5 (14–30)	28 (15–30)	0.002

Legend: ESII: EuroSCORE II; STS M: Society of Thoracic Surgeons Mortality; STS MM: Society of Thoracic Surgeons Morbimortality; EFS: Edmonton Frail Scale; GDS 30: Geriatric Depression Scale 30 items; GDS15: Geriatric Depression Scale 15 items; MMSE: Mini-Mental State Examination.

**Table 4:** Univariable Cox regression results of the candidate variables to the multivariable analysis.

		95% Confidence Interval		
Variables	HR	Lower Bound	Upper Bound	*p*-Value
ESII	1.304	1.103	1.541	0.002
STS M	1.219	1.020	1.456	0.029
STS MM	1.046	1.006	1.088	0.025
EFS binary (Fit to Moderate Frailty vs. Severe Frailty)	3.411	1.459	7.971	0.005
GDS30 binary (Normal to Mild Depression vs. Severe Depression)	3.584	1.069	12.020	0.039
Social Support	0.234	0.080	0.685	0.008
CPB duration	1.011	1.006	1.016	<0.001
Cross-Clamping duration	1.013	1.006	1.020	<0.001
Intraoperative Adverse Events	8.479	1.991	36.104	0.004
Delirium	2.100	0.921	4.790	0.078
Re-Operation *	5.807	1.731	19.483	0.004
Pneumonia *	4.050	1.207	13.585	0.024
Sternal infection *	8.867	2.080	37.794	0.003
Renal Failure *	3.761	1.121	12.618	0.032
Myocardial Infarction *	3.261	0.440	24.149	0.247
Composite of Adverse Events at 30 days *	3.212	1.374	7.507	0.007
Re-Operation up to 365 days	3.929	1.467	10.526	0.006

Legend: ESII: EuroSCORE II; STS M: Society of Thoracic Surgeons Mortality; STS MM: Society of Thoracic Surgeons Morbimortality; EFS: Edmonton Frail Scale; GDS 30: Geriatric Depression Scale 30 items; CPB: Cardiopulmonary Bypass; * as defined in STS version 2.61 Appendix A.

**Table 5:** Models results for survival determinants.

		95% Confidence Interval		
Models	HR	Lower Bound	Upper Bound	*p*-Value
1				
ESII	1.261	1.067	1.492	0.007
Pneumonia	5.353	1.558	18.388	0.008
Re-Intervention up to 365 days	3.159	1.095	9.113	0.033
2				
ESII	1.266	1.069	1.499	0.006
Pneumonia	4.192	1.169	15.034	0.028
Re-Intervention up to 365 days	3.143	1.091	9.056	0.034
Social Support	0.239	0.079	0.727	0.012

Legend: HR: Hazard Ratio; ESII: EuroSCORE II.

## Data Availability

According to EU legislation, health data are considered sensitive data, and were anonymized for this study. All data are available upon request to the corresponding author.

## Supplementary Materials

The supporting information can be downloaded at: https://www.mdpi.com/article/10.3390/jcm12144702/s1.

## Appendix A. STS Version 2.61 Definitions for Postoperative Outcomes

Complications: Indicate whether a postoperative event occurred during the hospitalization for surgery. This includes the entire postoperative period up to discharge, even if over 30 days.

Myocardial Infarction (MI):

Indicate the presence of a MI as documented by the following criteria:

- MI (0–24 h post-op): The CK-MB (or CK if MB not available) must be greater than or equal to 5 times the upper limit of normal, with or without new Q waves present in two or more contiguous ECG leads. No symptoms required.
- MI (>24 h post-op): Documented by at least one of the following criteria:

1. Evolutionary ST-segment elevations

2. Development of new Q-waves in two or more contiguous ECG leads

3. New or presumably new LBBB pattern on the ECG

4. The CK-MB (or CK if MB not available) must be greater than or equal to 3 times the upper limit of normal.

Reoperations (cardiac): Operative re-intervention was required for bleeding/tamponade, valvular dysfunction, graft occlusion and or other complications.

Sternum infection: Indicate whether the patient, within 30 days postoperatively, had a deep sternal infection involving muscle, bone, and/or mediastinum REQUIRING OPERATIVE INTERVENTION. Must have ALL of the following conditions:

1. Wound opened with excision of tissue (I&D) or re-exploration of mediastinum

2. Positive culture

3. Treatment with antibiotics

Pneumonia: Indicate whether the patient had Pneumonia diagnosed by any of the following:

Positive cultures of sputum, transtracheal fluid, bronchial washings, and/or clinical findings consistent with the diagnosis of pneumonia (which may include chest x-ray diagnostic of pulmonary infiltrates).

Renal failure: Indicate whether the patient had acute or worsening renal failure resulting in one or more of the following:

1. Increase of serum creatinine to >2.0, and 2× most recent preoperative creatinine level.

2. A new requirement for dialysis postoperatively.

Stroke: Indicate whether the patient has a postoperative stroke (i.e., any confirmed neurological deficit of abrupt onset caused by a disturbance in cerebral blood supply) that did not resolve within 24 h.
