# Supplementary material for: One-Year Survival after Cardiac Surgery in Frail Older People—Social Support Matters: A Prospective Cohort Study
Source: J Clin Med. 2023 Jul 15;12(14):4702. doi: 10.3390/jcm12144702 (PMC10381118; doi:10.3390/jcm12144702)
Supplement: Supplementary file 1 [file jcm-12-04702-s001.zip › jcm-2495578-supplementary.pdf]

GDS 15

Geriatric Depression Scale (15 itens)

Yesavage et al (1983)

Nome : ___________________________________ **Idade :** _____ **Data:** ____________

Responda **Sim** ou **Não** consoante se tem sentido de há uma semana para cá:

	Sim	Não
1. Está satisfeito com a sua vida?	**S**	**N**
2. Pôs de lado muitas das suas actividades e interesses?	**S**	**N**
3. Sente a sua vida vazia?	**S**	**N**
4. Fica muitas vezes aborrecido(a)?	**S**	**N**
5. Está bem disposto(a) a maior parte do tempo?	**S**	**N**
6. Tem medo que lhe vá acontecer alguma coisa de mal?	**S**	**N**
7. Sente-se feliz a maior parte do tempo?	**S**	**N**
8. Sente-se muitas vezes desamparado(a)?	**S**	**N**
9. Prefere ficar em casa, em vez de sair e fazer coisas novas?	**S**	**N**
10. Acha que tem mais dificuldades de memória do que as outras pessoas?	**S**	**N**
11. Pensa que é bom estar vivo(a)?	**S**	**N**
12. Sente-se inútil?	**S**	**N**
13. Sente-se cheio(a) de energia?	**S**	**N**
14. Sente que para si não há esperança?	**S**	**N**
15. Pensa que a maioria das pessoas passa melhor que o(a) senhor(a)?	**S**	**N**

Pontuação:
0-4 normal
5-8 ligeiro
9-11 moderado
12 -15 acentuado

> 5 pontos é sugestivo de Depressão (explorar através de uma avaliação mais completa)
≥ 10 pontos é quase sempre indicativo de depressão

Nota: esta escala foi concebida para auto-avaliação.
O autor considera que a escala está no domínio público

ESCALA DE DEPRESSÃO GERIÁTRICA

GERIATRIC DEPRESSION SCALE (GDS)

Nome: ____

Idade: ______ Data de Nascimento: __/__/__

Responda Sim ou Não consoante se tem sentido de há uma semana para cá:

	Sim	Não
1. Está satisfeito(a) com a sua vida?		
2. Pôs de lado muitas das suas actividades e interesses?		
3. Sente a sua vida vazia?		
4. Fica muitas vezes aborrecido(a)?		
5. Tem esperança no futuro?		
6. Anda incomodado(a) com pensamentos que não consegue afastar?		
7. Está bem disposto(a) a maior parte do tempo?		
8. Tem medo que lhe vá acontecer alguma coisa de mal?		
9. Sente-se feliz a maior parte do tempo?		
10. Sente-se muitas vezes desamparado(a)?		
11. Fica muitas vezes inquieto(a)? e nervoso(a)?		
12. Prefere ficar em casa, em vez de sair e fazer coisas novas?		
13. Preocupa-se muitas vezes com o futuro?		
14. Acha que tem mais problemas de memória do que as outras pessoas?		
15. Pensa que é bom estar vivo(a)?		
16. Sente-se muitas vezes desanimado(a) e abatido(a)?		
17. Sente-se inútil?		
18. Preocupa-se muito com o passado?		
19. Acha a vida interessante?		
20. É difícil para si começar novas actividades?		
21. Sente-se cheio(a) de energia?		
22. Sente que para si não há esperança?		
23. Pensa que a situação da maioria das pessoas passa é melhor do que a sua?		
24. Aflige-se muitas vezes com pequenas coisas?		
25. Sente muitas vezes vontade de chorar?		
26. Tem dificuldade em se concentrar?		
27. Gosta de se levantar de manhã?		
28. Prefere evitar encontrar-se com muitas pessoas?		
29. Tem facilidade em tomar decisões?		
30. O seu pensamento é tão claro como era antes?		
TOTAL		

Pontuação da GDS de 30 itens:

1 ponto para as respostas Sim nas questões: 2-4, 6, 8, 10-14, 16-18, 20, 22-26, 28

1 ponto para as respostas Não nas questões: 1, 5, 7, 9, 15, 19, 21, 27, 29, 30

0-10 = ausência de depressão; 11-20 = depressão ligeira; 21-30 = depressão grave

Escala de Fragilidade de Edmonton

Versão de Cabeceira

Data	B	x 1 =
Examinador	C	x 2 =
(nome, relação com o paciente)<br>Fonte Adicional	Pontuação Total	

IDENTIFICAÇÃO DO PACIENTE

Pontuar a EFE	FORMA	VULNERÁVEL	LIGEIRA	MODERADA	SEVERA
	1 2 3	4 5	6 7	8 9	10 +
			FRAGILIDADE		

Questões	A	B	C
Para cada item escolha apenas uma opção na coluna A, B ou C. A pontuação é atribuída com base na coluna. Por favor veja o Kit da Ferramenta EFE para instruções detalhadas	A=0	B=1	C=2
**1.Cognição**			
**Teste do Relógio**<br>"Imagine que o círculo desenhado é um relógio. Coloque os números **na** posição correta e de seguida marque "onze horas e dez".	PASSA	FALHA COM ERROS MINOR	FALHA COM ERROS MAIOR

Dobre pelo pontilhado de modo a evitar distrações antes de pedir ao doente para começar

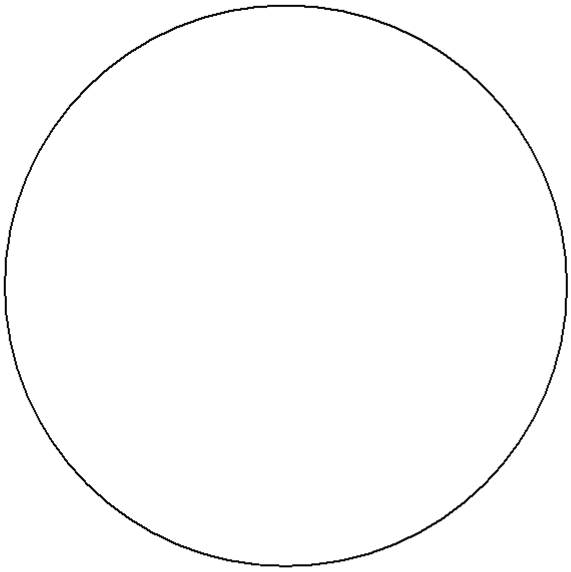

Escala de Fragilidade de Edmonton

Versão de Cabeceira

Questões	A	B	C
Se o Teste do Relógio (item 1) pontua na coluna B ou C, então os itens seguintes marcados com asterisco podem pontuar com base na melhor informação disponível*	A=0	B=1	C=2
**2. Estado Geral de Saúde**			
*Durante o último ano quantas vezes é que foi internado num hospital?	0	1-2	>2
Em geral como é que sente que está a sua saúde? (Escolha uma)	EXCELENTE<br>MUITO BOA<br>BOA	RAZOÁVEL	MÁ
**3. Independência Funcional**			
*Para quais das seguintes atividades **precisa** de ajuda?<br>☐ Preparar refeições ☐ Ir às compras ☐ Telefonar ☐ Limpar a casa<br>☐ Tomar medicação ☐ Usar transportes ☐ Tratar da roupa ☐ Governar dinheiro	0-1	2-4	5-8
**4. Suporte Social**			
Quando necessita de ajuda, pode contar com alguém que está disposto e é capaz de satisfazer as suas necessidades?	SEMPRE	ÀS VEZES	NUNCA
**5. Utilização de Medicamentos**			
*Toma cinco ou mais medicamentos diferentes regularmente?	NÃO	SIM	
*Às vezes esquece-se de tomar os medicamentos que lhe receitaram?	NÃO	SIM	
**6. Nutrição**			
*Perdeu peso recentemente de tal modo que a sua roupa lhe ficou mais larga?	NÃO	SIM	
**7. Humor**			
Costuma sentir-se frequentemente triste ou deprimido?	NÃO	SIM	
**8. Continência**			
*Perde urina sem querer?	NÃO	SIM	
**9. Desempenho Funcional**			
**Teste Timed Get Up and Go – 3 metros**<br>"Sente-se agora nesta cadeira, com as costas e braços apoiados. Depois, quando eu disser "ANDE" levante-se e ande no seu passo habitual até à marca que está no chão, e depois volte para a cadeira e sente-se."<br><br>**Tempo total registado** ___________ segundos<br><br>*Pontue este item >20 segundos se :*<br>a)paciente relutante ou incapaz de completar o teste<br>b)a execução segura do teste requer um auxiliar da marcha ou a ajuda de outra pessoa	0-10<br>SEGUNDOS	10-20<br>SEGUNDOS	>20<br>SEGUNDOS

Escala de Fragilidade de Edmonton – Versão de Cabeceira Oficial © 2019 Universidade de Alberta. Todos os direitos reservados. Estes materiais não podem ser copiados, publicados, traduzidos, distribuídos ou reproduzidos sob qualquer forma, no todo ou em parte sem uma licença da Universidade de Alberta.
Baseada na versão original © 2000 apresentada na Reunião Científica Anual da Sociedade Canadiana de Geriatria, posteriormente publicada em formato abreviado em Rolfson DB, et al, Validity and reliability of the Edmonton Frail Scale, Age and Ageing 2006 ; 35(5): 526-529 doi : 10.1093/ageing/afl041.
